# Sex differences in left atrial volumes, mechanics, and stiffness in primary mitral regurgitation—a combined 2D and 3D echocardiographic study

**DOI:** 10.1093/ehjci/jeae072

**Published:** 2024-03-12

**Authors:** Christian E Berg-Hansen, Rasmus Bach Sindre, Lisa M D Grymyr, Barbara Rogge, Andreas E Valeur, Stig Urheim, Judy Hung, Dana Cramariuc

**Affiliations:** Department of Heart Disease, Haukeland University Hospital, Jonas Lies vei 65, NO-5021 Bergen, Norway; Department of Clinical Science, University of Bergen, Jonas Lies vei 87, NO-5021 Bergen, Norway; Department of Clinical Science, University of Bergen, Jonas Lies vei 87, NO-5021 Bergen, Norway; Department of Heart Disease, Haukeland University Hospital, Jonas Lies vei 65, NO-5021 Bergen, Norway; Department of Clinical Science, University of Bergen, Jonas Lies vei 87, NO-5021 Bergen, Norway; Department of Heart Disease, Haukeland University Hospital, Jonas Lies vei 65, NO-5021 Bergen, Norway; Department of Heart Disease, Haukeland University Hospital, Jonas Lies vei 65, NO-5021 Bergen, Norway; Department of Clinical Science, University of Bergen, Jonas Lies vei 87, NO-5021 Bergen, Norway; Department of Heart Disease, Haukeland University Hospital, Jonas Lies vei 65, NO-5021 Bergen, Norway; Department of Clinical Science, University of Bergen, Jonas Lies vei 87, NO-5021 Bergen, Norway; Division of Cardiology, Cardiac Ultrasound Laboratory, Massachusetts General Hospital, Harvard Medical School, 55 Fruit St, Boston, MA 02114, USA; Department of Heart Disease, Haukeland University Hospital, Jonas Lies vei 65, NO-5021 Bergen, Norway; Department of Clinical Science, University of Bergen, Jonas Lies vei 87, NO-5021 Bergen, Norway

**Keywords:** mitral regurgitation, left atrial stiffness, left atrial strain, 3D echocardiography, mitral valve

## Abstract

**Aims:**

Mitral regurgitation (MR) causes left atrial (LA) enlargement and impaired reservoir function. We assessed whether changes in LA size, strain, and stiffness in significant (moderate or greater) primary MR are sex-specific.

**Methods and results:**

In the 3D Echocardiography and Cardiovascular Prognosis in Mitral Regurgitation study, 111 patients with primary MR were prospectively investigated with 2D and 3D echocardiography. MR was severe if the 3D regurgitant fraction was ≥50%. LA size was assessed by maximum, minimum, and pre-A 3D volume (LAV), mechanics by peak reservoir (LASr) and contractile strain, and stiffness by the ratio: mitral peak E-wave divided by the annular e′ velocity (E/e′)/LASr. Women were older, had higher heart rate, and lower body mass index and MR regurgitant volumes (*P* < 0.05). 3D LAV indexed for body surface area and LA contractile strain did not differ by sex, while LASr was lower (22.2 vs. 25.0%) and LA stiffness higher in women (0.56 vs. 0.44) (*P* < 0.05). In linear regression analysis, female sex was associated with higher LA stiffness independent of age, minimum LAV, left ventricular global longitudinal strain, diabetes, and coronary artery disease (*R*^2^ = 0.56, all *P* < 0.05). In logistic regression analysis, women had a four-fold (95% CI 1.2–13.1, *P* = 0.02) higher adjusted risk of increased LA stiffness than men.

**Conclusion:**

Women with significant primary MR have more impaired LA reservoir mechanics and increased LA stiffness compared with men despite lower MR regurgitant volumes and similar indexed LA size. The findings reveal sex-specific features of LA remodeling in MR.

**Trial Registration:**

ClinicalTrials.gov Identifier: NCT04442828

## Graphical Abstract

**Graphical Abstract:**
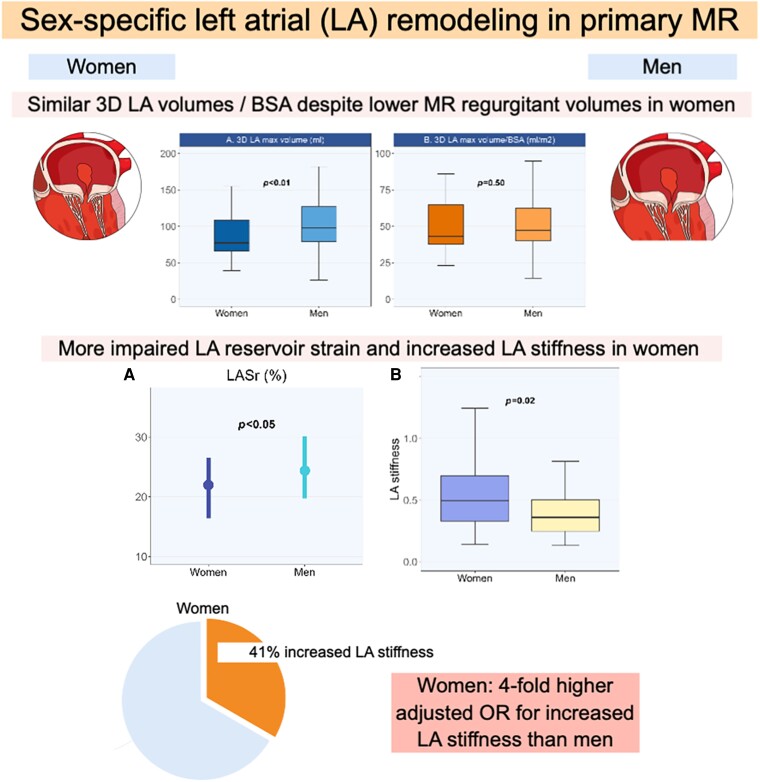
Sex-specific LA remodeling in primary MR. BSA, body surface area; LA, left atrial; LASr, left atrial reservoir strain; MR, mitral regurgitation; OR, odds ratio.

## Introduction

Chronic mitral regurgitation (MR) causes complex structural and functional changes in the left atrium including a progressive increase in size and interstitial fibrosis.^1^ Maximum left atrial (LA) size has been regarded as the hallmark of LA remodeling and is the only LA-related guideline-recommended criterion for grading MR severity.^2^ However, among patients with primary MR, significantly fewer women than men reach the proposed universal cut-off for severe LA dilatation of 60 mL/m^2^, and excess mortality occurs at a much smaller LA size.^3^

Recent research has focused on changed LA mechanics in MR and showed that impaired LA reservoir function predicts higher mortality in both primary and functional MR independent of the LA size.^4–6^ These findings suggest that not the LA size alone but rather a combined assessment of LA size and function should be part of the risk assessment in chronic MR. Whether changes in LA volumes and mechanics in primary MR differ in women and men has so far been scarcely explored.

Accumulating interstitial fibrosis in the LA wall in response to chronic stretch is thought to play a key role in impaired LA mechanics. However, this has been little investigated. In chronic LA pressure overload due to hypertrophic obstructive cardiomyopathy, women were shown to have increased susceptibility to myocardial fibrosis and lower atrial emptying rate.^7^ In 46 patients with primary MR, low LA reservoir strain (LASr) was found to be associated with increased LA fibrosis at histopathological examination.^8^ LA stiffness, as derived non-invasively from the ratio of early diastolic mitral inflow velocity and mitral annular early diastolic velocity, i.e. the mitral E/e′, and LASr, has not been previously reported in primary MR.^9^

In the current study, we assessed whether moderate or greater primary MR causes sex-specific changes in (i) 3D LA volumes; (ii) reservoir and contractile LA mechanics; and (iii) LA stiffness. Moreover, we analysed the relation between increased LA stiffness and traditional cardiovascular (CV) risk factors as well as left ventricular (LV) mechanics, mitral valve geometry, and pulmonary vein haemodynamics in patients with primary MR.

## Methods

### Study population

The 3D Echocardiography and Cardiovascular Prognosis in Mitral Regurgitation (3D-PRIME) study is a prospective follow-up of patients aged ≥18 years with significant (moderate or greater) MR and no other severe valvular heart disease referred to the Heart Valve Clinic at Haukeland University Hospital. At inclusion, all patients undergo clinical examination, electrocardiography (ECG) and combined 2D and 3D transthoracic and transoesophageal echocardiography. Patients with 3D echo acquisitions of low resolution as well as those with hypertrophic cardiomyopathy, congenital heart disease, pulmonary embolism, or constrictive pericarditis are excluded.

In the present analysis, we used baseline data collected in the first 185 patients recruited in the 3D-PRIME study between 2020 and 2023. Of these, 111 had primary MR and thus constituted the present study population. 3D minimum (LAVmin), pre-A (LAVpreA), and maximum LA (LAVmax) volumes as well as the 3D LA emptying fraction were analysed in the whole cohort. Analyses of 2D LA strain and stiffness were restricted to 99 patients in sinus rhythm at the echocardiographic examination.

In all patients, data on symptoms, comorbidities, and medical treatment were collected through patient interviews at the study visits and chart review. Hypertension was defined as a history of hypertension, blood pressure (BP) ≥ 140/90 mmHg at the study visit, and/or use of BP-lowering medication. The presence of atrial fibrillation at examination as well as previous episodes of atrial fibrillation documented in the medical charts was reviewed in each patient. The study was conducted in accordance with the revised Declaration of Helsinki. All patients gave written informed consent, and the study was approved by the regional ethics committee (2020/106848).

### Echocardiographic measurements

Comprehensive 2D and 3D transthoracic and transoesophageal echocardiograms were performed using commercially available ultrasound equipment (Vivid E95, GE Vingmed Ultrasound, Horten, Norway). All data were analysed offline by a junior and proofread by a senior reader. Analyses were performed on Echopac workstations (version 204, GE Vingmed Ultrasound, Horten, Norway) equipped with both 2D speckle tracking software and specific 3D software (4D Auto LAQ, LVQ, and MVQ) for semi-automatic quantification of the LA and LV 3D volumes and function as well as of mitral valve 3D geometry.

#### LA volumes, mechanics, and stiffness

LA volumes were measured first in 2D using apical four- and two-chamber-dedicated LA acquisitions, and then in four- to six-beat full-volume 3D acquisitions with an average temporal resolution of 59 ± 19 vol/s.^10^ Both in 2D and 3D, LA volumes were measured at end-diastole (the frame just after mitral valve closure), onset of atrial contraction (identified by the P-wave on ECG) and end-systole (the frame just before mitral valve opening), rendering LAVmin, LAVpre-A, and LAVmax, respectively.^11^ LA appendage and the ostia of pulmonary veins were excluded from the LA volumes. In 3D acquisitions, the interface blood tissue identified by the software in each apical view was manually checked on each frame and adjusted by the reader where needed.

All LA volumes are reported as absolute values as well as values indexed for body surface area (BSA). LA dilatation was defined as either 3D LAVmax/BSA ≥ 43 mL/m^2^ or 3D LAVmin/BSA ≥ 18 mL/m^2^.^12^ The LA emptying fraction was calculated as the ratio: (LAVmax − LAVmin)/LAVmax.

LA strain was measured in 2D acquisitions using specific software (AFI LA), and end-diastole as the zero-strain reference point.^13^ In patients in sinus rhythm, LA reservoir function was assessed by the reservoir strain (LASr) and the reservoir work (LAWr), and LA pump function by the contractile strain. LA reservoir work was deducted from the product LASr × LA reservoir volume as previously described.^5^

The early diastolic transmitral inflow velocity (E) was measured in pulsed-wave Doppler acquisitions. Peak mitral annulus velocity during early diastole (e′) was measured by tissue Doppler imaging in both the septum and lateral wall and averaged. The mitral E/e′ ratio was used as a surrogate of mean LA pressure based on previously demonstrated good agreement with LA pressure in functional MR as well as in patients with significant primary MR.^14,15^ LA stiffness was derived from the mitral E/e′ divided by LASr and classified as high for age if ≥0.22 in patients aged <40 years, ≥ 0.42 in those ≥40 and <60 years, and ≥0.55 in patients ≥60 years old.^9,16^

#### Mitral valve geometry and MR grading

Mitral valve geometry was quantified using four- to six-beat full-volume 3D zoomed mitral data sets with an average resolution of 42 ± 19 vol/s.^17^ For the present analysis, mitral valve remodeling was assessed by the antero-posterior and anterolateral-posteromedial annular diameter, the anterior and posterior leaflet area, and the total annular area at mid-systole. MR was assessed by an integrated approach based on qualitative, semiquantitative, and quantitative parameters.^2,18^ Quantification included the proximal isovelocity surface area-based effective regurgitant orifice area (EROA), the regurgitant volume, and the 3D regurgitant fraction (the ratio of MR volume to the 3D LV total stroke volume).^2,18^ A regurgitant fraction ≥50% was considered indicative of severe MR.

#### Pulmonary vein flow

At transeosophageal echocardiography, the systolic pulmonary vein velocity time integral was measured in the upper right and upper left pulmonary vein and averaged.^19^

#### LV size and function

3D LV end-diastolic and end-systolic volumes were measured in four- to six-beat full-volume transthoracic 3D datasets with an average resolution of 40 ± 8 vol/s.^20,21^ LV global systolic function was assessed by the 3D ejection fraction (EF) and the 2D global longitudinal strain (GLS) measured in all three standard apical views.^22^

### Statistical analyses

Statistical analyses were performed in IBM SPSS Statistics 28.0 (IBM Corp., Armonk, NY, USA) as well as R version 4.2.3 (R Foundation for Statistical Computing, Vienna, Austria). LA volumes, strain, and stiffness were compared between women and men, and between patients with severe vs. non-severe MR. Moreover, LASr values adjusted for differences in clinical characteristics and for LA size were compared between women and men using a general linear model with the Bonferroni test of estimated marginal means. Findings are reported as mean ± standard deviation, median, and interquartile range (IQR) or percentages. Comparisons between groups were performed by *χ*^2^ tests, independent-samples *t*-tests, or Mann–Whitney *U* test, as appropriate.

The association between LA stiffness and clinical and echocardiographic variables was tested in Spearman rank-order tests, as well as in univariable and multivariable regression analyses. Covariates in the multivariable models were included based on clinical relevance and association with LA stiffness at *P* < 0.1 in the univariable models. The multivariable linear regression model was run with a backward stepwise procedure with stepwise elimination of the non-significant variables among age, sex, body mass index (BMI), heart rate, systolic BP, diabetes, coronary artery disease, chronic kidney disease, CV medication, MR regurgitant volume, mitral anterolateral-posteromedial diameter, LAVmin, and LV GLS. The final model is presented using standardized *β* coefficients and *P*-values of significance. The same variables included in the linear regression model were additionally tested in relation to increased LA stiffness for age in a multivariable logistic regression analysis run with a backward stepwise procedure. A two-tailed *P* < 0.05 was considered significant in all analyses.

## Results

### Clinical characteristics

At the study baseline, women (34% of the cohort) were older and had higher heart rate, but lower BMI and diastolic BP than men (*Table 1*). The prevalence of traditional CV risk factors and coronary artery disease, as well as use of antihypertensive, antidiabetic, and lipid-lowering medication were similar between sexes. In total, 89% of the patients were in sinus rhythm at the echocardiographic examination (*Table 1*).

**Table 1:** Clinical characteristics of the whole study population and separately of women and men with significant primary mitral regurgitation

	All	Women	Men	*P*-value
	(*n* = 111)	(*n* = 38)	(*n* = 73)	
Age, years	68 (56–78)	74 (61–80)	64 (54–75)	0.013
BMI, kg/m^2^	25.3 ± 3.5	24.3 ± 3.9	25.9 ± 3.2	0.040
Heart rate, bpm	67 (60–76)	72 (65–78)	64 (59–74)	0.007
Systolic BP, mmHg	136 (124–153)	142 (126–162)	136 (124–148)	0.233
Diastolic BP, mmHg	81 (75–88)	77 (70–86)	82 (76–89)	0.045
Atrial fibrillation at examination	11%	8%	12%	0.475
History of paroxysmal atrial fibrillation	18%	18%	19%	0.923
History of hypertension	41%	45%	38%	0.516
Diabetes mellitus	2%	3%	1%	0.635
Coronary artery disease	17%	11%	21%	0.183
Chronic kidney disease	8%	11%	7%	0.501
Medication:				
Antihypertensive	58%	63%	55%	0.397
Lipid-lowering	37%	40%	36%	0.690
Antidiabetic	2%	3%	1%	0.635

Data are presented as median (IQR), mean ± standard deviation, or as percentages.

The *P*-values in the last column are based on independent-samples *t*-tests for normally distributed variables and Mann–Whitney *U* tests for not normally distributed variables.

BMI, body mass index; BP, blood pressure; IQR, interquartile range.

### LA size and mechanics

**Figure 1:**
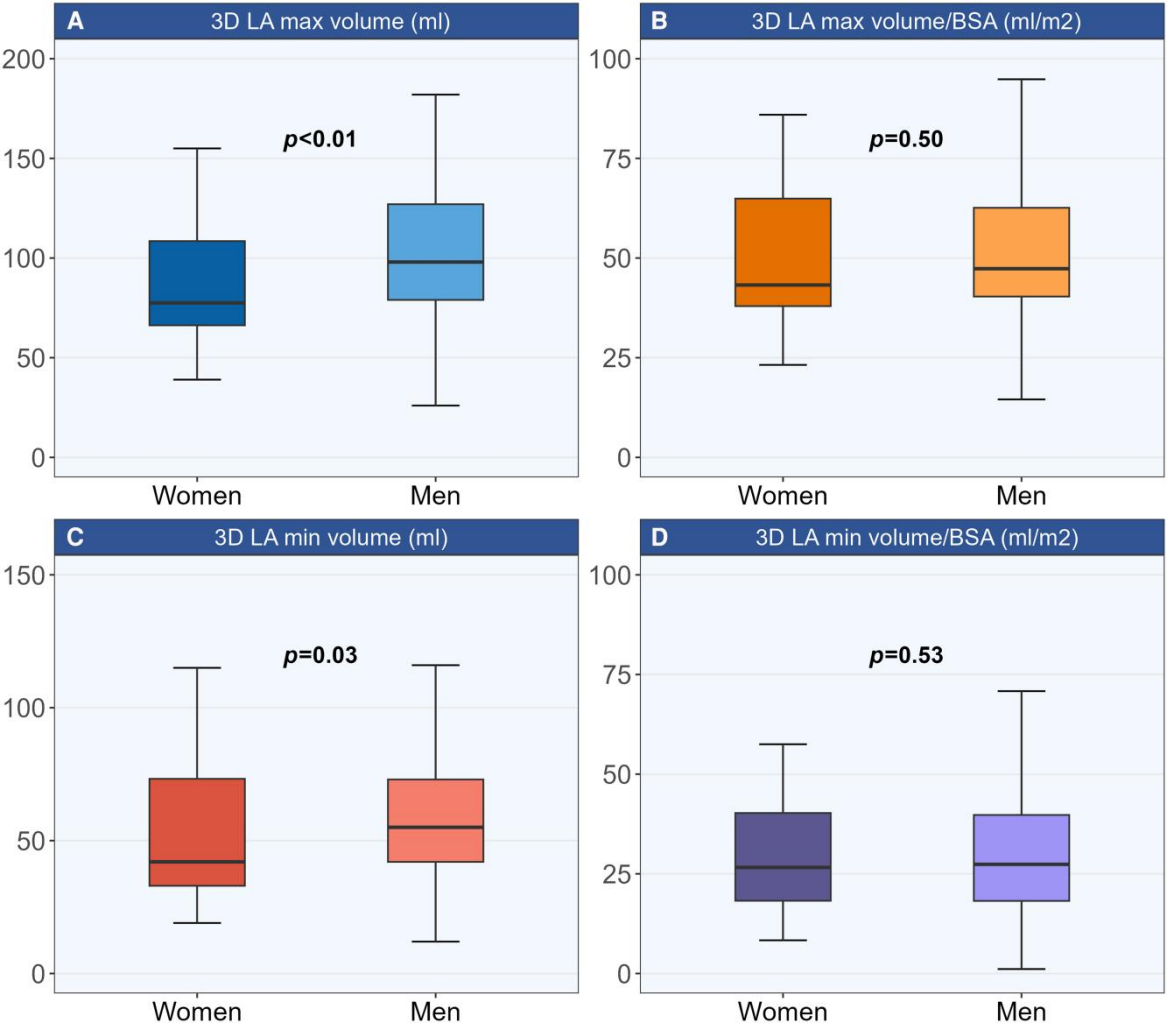
3D left atrial volumes in women and men with primary mitral regurgitation. (*A*) 3D maximum left atrial volume; (*B*) 3D maximum left atrial volume indexed for body surface area; (*C*) 3D minimum left atrial volume; (*D*) 3D minimum left atrial volume indexed for body surface area. The *P*-values are based on the Mann–Whitney *U* test. BSA, body surface area; LA, left atrial.

Severe MR was present in 56% of the population and comparably frequent in women and men: 50 vs. 59% (*P* = 0.36). MR EROAs and volumes were significantly lower in women, but due to proportionally lower LV stroke volumes, the MR regurgitation fraction was similar between sexes (*Table 2*). 3D LA volumes were lower in women in absolute value, but similar to those in men after indexation for BSA (*Figure 1*, *Table 2*). A dilated LA defined as increased LAVmax/BSA was present in 61% of patients and significantly more common in severe (77%) than in non-severe MR (42%) (*P* < 0.001). When dilatation was defined as increased LAVmin/BSA, 84% of patients (92% of those with severe and 73% of those with non-severe, *P* = 0.008) had increased LA size. The prevalence of LA dilatation by either LAVmax or LAVmin indexed for BSA was comparable between sexes.

**Table 2:** Echocardiographic characteristics of the whole study population and separately of women and men with significant primary mitral regurgitation

	All	Women	Men	*P*-value
	(*n* = 111)	(*n* = 38)	(*n* = 73)	
LA volumes and function				
2D LAVmax, mL	91 (72–127)	81 (58–112)	99 (74–131)	0.014
2D LAVmax/BSA, mL/m^2^	47 (37–65)	46 (35–64)	49 (37–66)	0.434
3D LAVpreA, mL	71 (56–95)	59 (49–95)	75 (61–96)	0.008
3D LAVpreA/BSA, mL/m^2^	36 (30–51)	34 (29–52)	38 (30–50)	0.559
3D LA emptying fraction, %	42 ± 12	42 ± 13	42 ± 12	0.933
LV volumes, function and filling pressure				
3D LV EDV, mL	162 (133–201)	130 (107–161)	183 (150–220)	<0.001
3D LV ESV, mL	65 (49–83)	49 (41–60)	73 (58–91)	<0.001
3D LV stroke volume, mL	98 (76–123)	80 (65–98)	106 (89–130)	<0.001
3D LVEF, %	59 (56–65)	60 (56–67)	59 (56–64)	0.322
LV GLS, %	−18.1 (−15.9–−20.4)	−18.2 (−15.8–−20.8)	−18.1 (−15.8–−20.2)	0.848
Mitral E/e′	11 ± 6	11 ± 5	10 ± 6	0.243
3D mitral valve geometry and haemodynamics				
Annulus area, cm^2^	14.2 ± 3.9	12.4 ± 3.7	15.2 ± 3.6	<0.001
Anterior leaflet area, cm^2^	7.2 ± 1.9	6.3 ± 1.9	7.6 ± 1.7	0.001
Posterior leaflet area, cm^2^	7.6 (5.8–10.0)	7.0 (4.8–8.1)	8.1 (6.4–10.8)	0.009
Antero-posterior diameter, cm	3.72 ± 0.55	3.48 ± 0.55	3.85 ± 0.51	0.001
Anterolateral-posteromedial diameter, cm	4.27 ± 0.64	3.97 ± 0.64	4.43 ± 0.58	<0.001
Mitral e′, cm/s	10 ± 3	10 ± 3	9 ± 3	0.020
Mitral E, cm/s	9.3 ± 0.3	9.3 ± 0.3	9.3 ± 0.3	0.999
MR quantification				
MR EROA, mm^2^	26 (17–63)	18 (15–38)	35 (21–73)	0.001
MR regurgitant volume, mL	49 (29–90)	34 (25–60)	67 (36–111)	0.006
MR regurgitant fraction, %	56 (35–84)	50 (33–77)	60 (37–91)	0.315

Data are presented as median (IQR), mean ± standard deviation, or as percentages.

The *P*-values in the last column are based on independent-samples *t*-tests for normally distributed variables and Mann–Whitney *U* tests for not normally distributed variables.

BSA, body surface area; EDV, end-diastolic volume; EF, ejection fraction; EROA, effective regurgitant orifice area; ESV, end-systolic volume; GLS, global longitudinal strain; IQR, interquartile range; LA, left atrial; LAV, left atrial volume; LV, left ventricular; MR, mitral regurgitation.

Similar to LA size, women had significantly smaller mitral valve annular diameters and area, and smaller anterior and posterior leaflet area than men. However, the differences in valve geometry were annulled by correction for BSA (*Table 2*).

**Figure 2:**
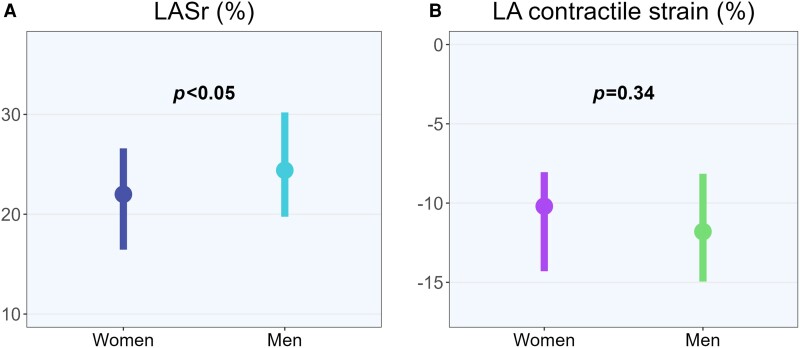
Left atrial function in women and men with primary mitral regurgitation. (*A*) Left atrial reservoir strain. (*B*) Left atrial contractile strain. The *P*-values are based on the Mann–Whitney *U* test. LA, left atrial; LASr, left atrial reservoir strain.

In patients in sinus rhythm, the LA reservoir mechanics assessed by either LASr or LAWr was lower in women than in men (LAWr: 823 in women vs. 1180 mlx% in men, *P* < 0.001). After adjustment for differences in age, heart rate, BMI, diastolic BP, and LAVmin, LASr was still significantly lower in women: 22.1 vs. 24.8% in men (*P* = 0.02). The 3D LA emptying fraction did not differ by sex (*Table 2*). LA pump function assessed by the LA contractile strain was comparable in women and men (*Figure 2*).

### LA stiffness

**Figure 3:**
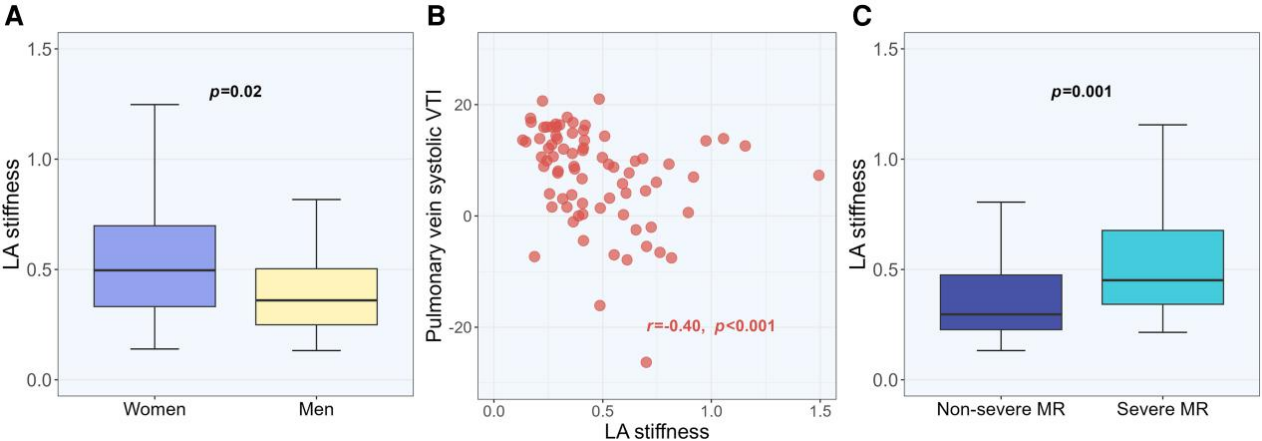
(*A*) Left atrial stiffness in women and men; (*B*) left atrial stiffness in relation to pulmonary vein systolic flow; (*C*) left atrial stiffness in severe vs. non-severe MR. The *P*-values are based on the Mann–Whitney *U* test (panels *A* and *C*) and the Spearman rank-order test (panel *B*). LA, left atrial; MR, mitral regurgitation; VTI; velocity time integral.

Increased LA stiffness for age was present in 32% of patients and more prevalent in women: 41 vs. 27% in men, and in patients with severe MR: 42 vs. 21% in non-severe MR (*Figure 3*). In the whole cohort, LA stiffness was associated with known hypertension (*r* = 0.32), diabetes (*r* = 0.24), coronary artery disease (*r* = 0.30), and chronic kidney disease (*r* = 0.25) (all *P* < 0.05). It also increased with age (*r* = 0.55) and higher systolic BP (*r* = 0.26), larger MR regurgitant volume (*r* = 0.24), larger LA volumes (in particular LAVmin, *r* = 0.52), and smaller mitral anterolateral-posteromedial diameter (*r* = −0.20) (all *P* < 0.05). LA stiffness was inversely associated with systolic pulmonary vein flow (*Figure 3*).

In multivariable linear regression analysis, after backward stepwise elimination of non-significant variables (BMI, heart rate, systolic BP, chronic kidney disease, CV medication, MR regurgitant volume, and mitral anterolateral-posteromedial diameter), female sex was related to increased LA stiffness independent of significant associations with older age, larger LAVmin, and lower LV GLS (*R*^2^ = 0.56, *Table 3*). When the same factors were tested in logistic regression analysis, women had a 3.96-fold (95% CI 1.20–13.05) higher risk of increased LA stiffness for age compared with men after adjustment for LV GLS, LAVmin, and the MR regurgitant volume (all *P* < 0.05) (Nagelkerke *R*^2^ = 0.45, *P* < 0.001).

**Table 3:** Factors independently associated with left atrial stiffness in multivariable linear regression analysis (*R*^2^ = 0.56, *P* < 0.001)

	Beta	*T*	*P*-value
Female sex	0.18	2.30	0.024
Age, years	0.21	2.58	0.012
Diabetes mellitus	0.14	1.73	0.088
Coronary artery disease	0.14	1.68	0.098
LAVmin, mL	0.41	5.37	<0.001
LV GLS, %	0.31	3.92	<0.001

LAVmin, minimum left atrial volume; LV GLS, left ventricular global longitudinal strain.

## Discussion

Retrospective analyses have shown that women with severe primary MR have smaller LA diameters and MR regurgitant volumes, but higher mortality and lower surgery rates than men.^3,23^ When operated, women develop more often postoperative heart failure.^3^ These observations suggest delayed diagnosis in women, among others due to the current MR grading system and smaller chamber sizes in women, and/or a sex-specific adverse cardiac remodeling with possibly more severe structural myocardial changes in women at similar or lower MR volumes. Despite these observations, current guidelines make no sex-specific recommendations in the grading or follow-up of patients with MR.^2,24^ Through comprehensive 2D and 3D echocardiographic profiling, we demonstrate that women with primary MR and lower regurgitant volumes than men with comparable regurgitant fraction have: (i) similar 3D LA volumes indexed for BSA; (ii) more impaired LA reservoir mechanics; and (iii) increased LA stiffness after adjustment for clinical differences, LA size, and LV GLS. Corroborated, the findings are consistent with a more advanced degree of atrial myopathy in women at lower MR regurgitant volumes.

Even if most previous research in MR used 2D LA diameters and volumes, the current expert recommendation is to assess LA size by 3D volumes to avoid geometric assumptions and volume underestimation.^25^ Earlier 3D echocardiographic studies showed that healthy women and men have similar LA volumes indexed for BSA.^12,26^ In severe primary MR, guidelines recommend mitral valve surgery for asymptomatic patients with no LV dysfunction but dilated LA (defined as above 55 mm or 60 mL/m^2^ at 2D echocardiography) despite sinus rhythm and if at low surgical risk.^2^ Our data support the use of a common cut-off for LA dilatation in both women and men if size is assessed by the 3D LA volumes indexed for BSA and as long as MR severity is graded based on the MR regurgitant fraction, as this better reflects the increased haemodynamic effect of a lower MR volume in the context of a lower forward stroke volume. Of note, despite mitral dimensions being significantly smaller in women than in men, current algorithms for assessing the mitral valve suitability for surgical or percutaneous repair use unindexed cut-offs for most of the valve geometric characteristics including the mitral ring size and leaflet length.^27^ Our data show that mitral dimensions become similar in men and women with significant primary MR after indexation for BSA and support this approach to avoid erroneous grading and under-referral of women for mitral intervention.

Analyses of LA mechanics from a cohort of 371 adults included in the NORRE study showed similar LASr values in healthy women and men with a median age of 45 (IQR 34–55) years.^16^ In patients with severe primary MR with indication for surgery, one-third of whom had atrial fibrillation, one previous analysis revealed a high prevalence of reduced LASr (≤22% in 52% of the cohort); however, sex-specific data were not reported in this study.^4^ In moderate and greater MR, we found that women had lower LA reservoir function at lower regurgitant volumes than men and despite similar LA volume index, suggesting a more advanced phase of atrial myopathy in women. Of note, LA emptying fraction did not differ by sex, but this is a rougher measure of LA reservoir function based solely on the relative difference between LAVmax and LAVmin and probably less susceptible to changes in atrial function than LASr. Interestingly, LA contractile strain was similar between sexes, possibly indicating impaired LA extensibility in the reservoir phase as a more sensitive marker of LA myopathy in women with MR. A pattern of reduced LASr with preserved contractile strain has previously been described in women from the general population with earlier stages of diastolic dysfunction, as well as in patients with hypertension or metabolic syndrome, but until now not in chronic MR.^28–30^

However, reduced LA extension in the reservoir phase can be due to a less compliant LA wall, but also to reduced preload. The cause of impaired strain can be further explored by assessment of LA stiffness, a non-invasive index that combines information about LA mechanics and filling pressures and has been previously validated against right heart catheterization.^9^ LA stiffness estimated from the ratio E/e′/LASr has recently been shown to be linked to a higher risk of death and heart failure hospitalizations and to have superior predictive abilities compared with traditional diastolic parameters in patients with heart failure with preserved EF.^31^ In patients with hypertension, increased LA stiffness has been proposed as an earlier marker of target-organ damage than LA dilatation.^32^ LA stiffness has to our knowledge not been previously assessed in patients with significant primary MR. A recent retrospective study in 72 dogs with myxomatous mitral valve disease showed that this index was associated with an increased risk of CV death and had a higher predictive value than LASr.^33^ In humans, we demonstrate that women with significant primary MR have increased LA stiffness compared with men despite lower MR volumes and after adjustment for differences in age and LA size. In univariate analyses, increased LA stiffness was related to the presence of traditional CV risk factors, including hypertension, in line with previous findings.^32^ However, after multiple adjustments for other covariates including BP and antihypertensive medication, female sex remained significantly associated with a higher risk of increased LA stiffness. This implies that women with MR might develop LA damage at lower volume overload than men or due to the concomitant involvement of other proinflammatory and profibrotic stimuli. Our finding should, however, be investigated further, among others by employing specific methods for fibrosis quantification.

Interestingly, LA stiffness was stronger related to minimum than to maximum LA size independent of LV GLS, reinforcing LAVmin as a possibly more relevant measure of LA remodeling than LAVmax.^34^

### Study limitations

The present study is a cross-sectional analysis of patients with significant MR and thus causal relationships cannot be inferred. LA stiffness at echocardiography is a newer method of estimating reduced LA wall compliance, and comparison with other imaging techniques has not yet been performed. The patients included in this analysis were selected among those referred for advanced diagnostics at one heart valve clinic, and the findings remain to be replicated in a larger cohort. LASr was 12% lower in women after adjustment for possible confounders, and the clinical relevance of this difference needs to be evaluated in relation to outcomes. Finally, LA stiffness has not been tested prospectively in patients with MR, and the thresholds associated with reduced survival in women and men as well as the incremental prognostic value of measuring LA stiffness to standard patient management in this clinical setting should be addressed by future studies.

## Conclusion

Women with significant primary MR have more impaired LA reservoir mechanics and increased LA stiffness compared with men after adjustment for differences in clinical characteristics, LA size and LV GLS. The findings support the combined assessment of LA size and function in the evaluation of patients with MR and highlight sex-specific mechanisms behind LA remodeling in primary MR.

## Data Availability

The data that support the findings of this study are available from the corresponding author upon reasonable request.
